# Two short low complexity regions (LCRs) are hallmark sequences of the Delta SARS-CoV-2 variant spike protein

**DOI:** 10.1038/s41598-022-04976-8

**Published:** 2022-01-18

**Authors:** Arturo Becerra, Israel Muñoz-Velasco, Abelardo Aguilar-Cámara, Wolfgang Cottom-Salas, Adrián Cruz-González, Alberto Vázquez-Salazar, Ricardo Hernández-Morales, Rodrigo Jácome, José Alberto Campillo-Balderas, Antonio Lazcano

**Affiliations:** 1 Facultad de Ciencias, Universidad Nacional Autónoma de México, 04510 Mexico City, Mexico; 2 Escuela Nacional Preparatoria, Plantel 8 Miguel E. Schulz, Universidad Nacional Autónoma de México, 01600 Mexico City, Mexico; 3 Department of Chemical and Biomolecular Engineering, University of California, Los Angeles, CA 90095 USA; 4 El Colegio Nacional, 06470 Mexico City, Mexico

**Keywords:** Molecular evolution, SARS-CoV-2, Viral evolution

## Abstract

Low complexity regions (LCRs) are protein sequences formed by a set of compositionally biased residues. LCRs are extremely abundant in cellular proteins and have also been reported in viruses, where they may partake in evasion of the host immune system. Analyses of 28,231 SARS-CoV-2 whole proteomes and of 261,051 spike protein sequences revealed the presence of four extremely conserved LCRs in the spike protein of several SARS-CoV-2 variants. With the exception of Iota, where it is absent, the Spike LCR-1 is present in the signal peptide of 80.57% of the Delta variant sequences, and in other variants of concern and interest. The Spike LCR-2 is highly prevalent (79.87%) in Iota. Two distinctive LCRs are present in the Delta spike protein. The Delta Spike LCR-3 is present in 99.19% of the analyzed sequences, and the Delta Spike LCR-4 in 98.3% of the same set of proteins. These two LCRs are located in the furin cleavage site and HR1 domain, respectively, and may be considered hallmark traits of the Delta variant. The presence of the medically-important point mutations P681R and D950N in these LCRs, combined with the ubiquity of these regions in the highly contagious Delta variant opens the possibility that they may play a role in its rapid spread.

## Introduction

Protein segments that exhibit a bias in their composition can be formed by (a) a small number of different amino acids, in which case they are called low complexity regions (LCRs); or (b) homopolymers or homorepeats, if they consist of a long repetition of a single amino acid^1,2^. LCRs tend to be more prevalent in proteins associated with polysaccharide-, ion-, and nucleic acid binding, as well as in phospholipid interaction, transcription, translation, and folding functions^3^. It is estimated that approximately 0.4% of eukaryotic proteomes are LCRs, which is up to 23 times higher than in prokaryotes^3^.

LCRs emergence has been associated with replication slippage and the formation of microsatellites during genome replication or recombination events^4,5^. The regions of the proteins in which the LCRs are located evolve rapidly, but there is an ongoing debate whether they change neutrally or under selective pressures^6^. Given the immunological significance of pathogens’ surface proteins in which many LCRs are located^5,7–10^, it is somewhat surprising that little attention has been given to their presence in viral proteomes. In sensu stricto, the presence and location of LCRs in viruses has only been reported in the HIV-1^9^ and, more recently, in SARS-CoV-2^11^. They are rather abundant in the HIV-1 gp120 protein, and over 30% of them are located in the hypervariable regions of the connecting loops present in the protein, where they may play a role in immune escape^9^. LCRs are scattered throughout the SARS-CoV-2 proteome, and are more prevalent in the non-structural protein 3, spike protein, and the nucleocapsid protein, where they may simultaneously enhance immune evasion and induce a strong immunogenic response^11^. However, they are conspicuously absent in several proteins of the replication-transcription complex (RdRp, helicase, and NSP14 exonuclease), and in the NSP1, 3CL protease, NSP9-11, NSP15, ORF3a, membrane (M) protein, ORF6, ORF8 and ORF10 proteins^11^.

In addition to LCRs, the presence of nucleotide simple sequence repeats (SSR) has also been reported in viral genomes. SSRs are DNA segments of tandemly repeated nucleotide motifs (e.g. di-, tri-, tetra, or penta-nucleotides) found in prokaryotic and eukaryotic genomes. Like LCRs, SSRs have also been associated with increased adaptability, as well as with enhanced recombination rates and indel generation in both cells and in viruses^12–15^. Viral SSRs are present in both RNA- and DNA viruses, including DNA mycobacteriophages^14^, economically relevant plant viruses such as potyviruses^16^, tobamoviruses^17^, and geminiviruses^18^, as well as in medically important viruses like herpesviruses^19^, HIV-1^20^ and filoviruses^21^. More recently, several SSRs rich in hexameric repeats have been identified in the SARS-CoV-2 genome^22,23^, which appear to be more prevalent in the ORF1ab, S, ORF3a, N and ORF7a of the SARS-CoV-2 genic regions^23^.

**Figure 1:**
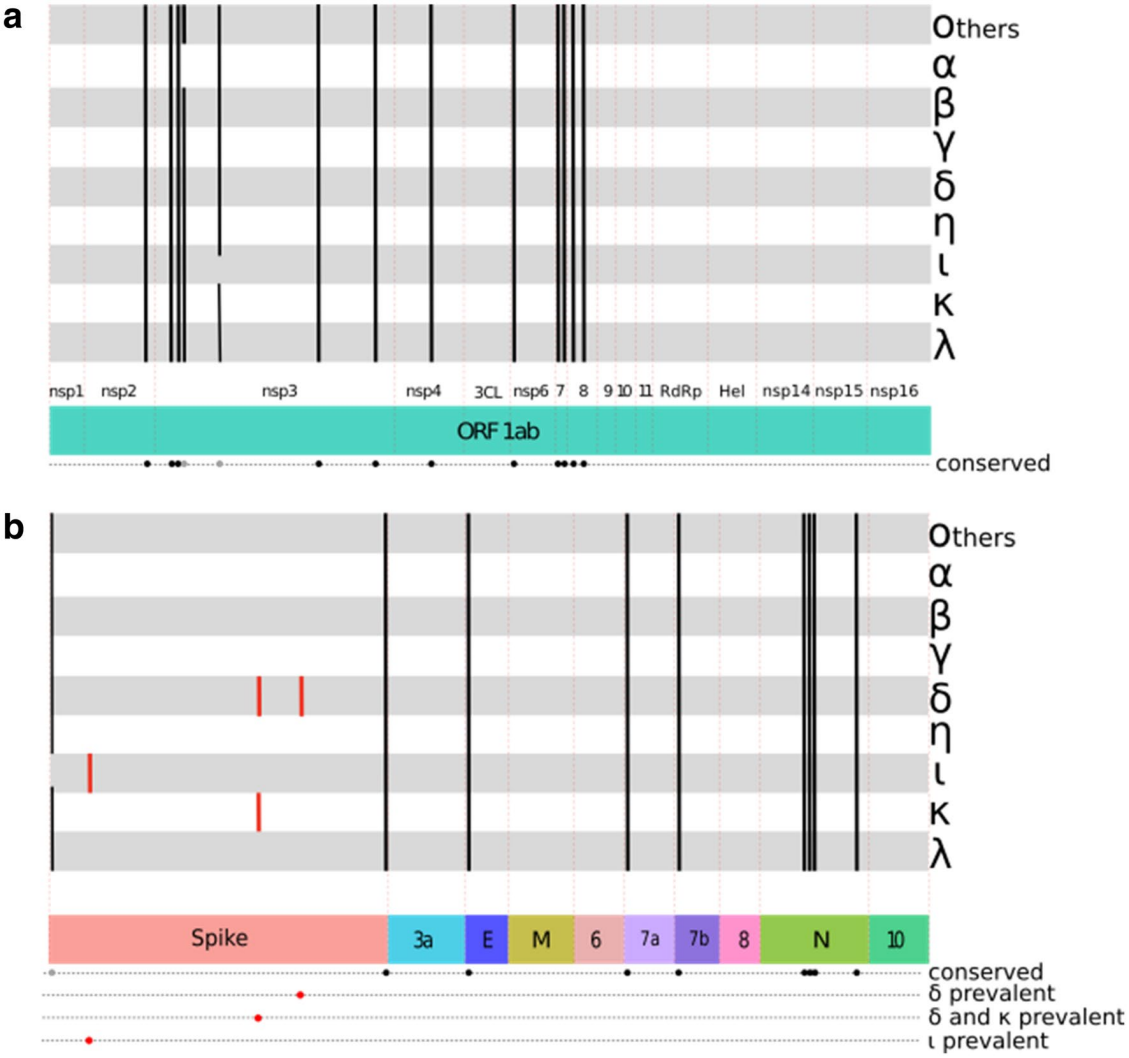
LCRs in VOCs (Alpha, Beta, Gamma and Delta variants), VOIs (Epsilon, Eta, Iota, Kappa and Lambda variants) and Other SARS-CoV-2 proteomes (Others). (**a**) LCRs present in ORF1ab, which includes nsp1-4, 3CL protease (3CL), nsp6-nsp11, RdRp polymerase (RdRp), Helicase (Hel) and nsp 14-16. (**b**) LCRs along spike, ORF3a (3a), envelope (E), membrane (M), ORFs 6, 7a, 7b, 8, nucleocapsid (N) and ORF10. The Spike LCR sequences reported here in the Delta, Iota and Kappa variants are represented with red lines. The width of the lines is not proportional to the number of sequences in each variant.

In this work, we report the conservation and variability of LCRs in several SARS-CoV-2 variants of concern (VOC) and interest (VOI) using comparative proteomics and protein structure analyses. We have identified three previously unreported LCRs that are present only in some VOIs and VOCs (Fig. 1). Quite significantly, these LCRs do not exhibit a random distribution in the proteins where they are located. Two of them are located in highly conserved positions of the spike S1 and S2 subunits of the extremely contagious Delta variant. Our results demonstrate that these two conserved (98–99%) short LCRs are hallmark sequences of the highly transmissible Delta SARS-CoV-2 variant, which suggest that they might play a significant role in the viral adaptation and rapid spread of this VOC.

## Results

**Figure 2:**
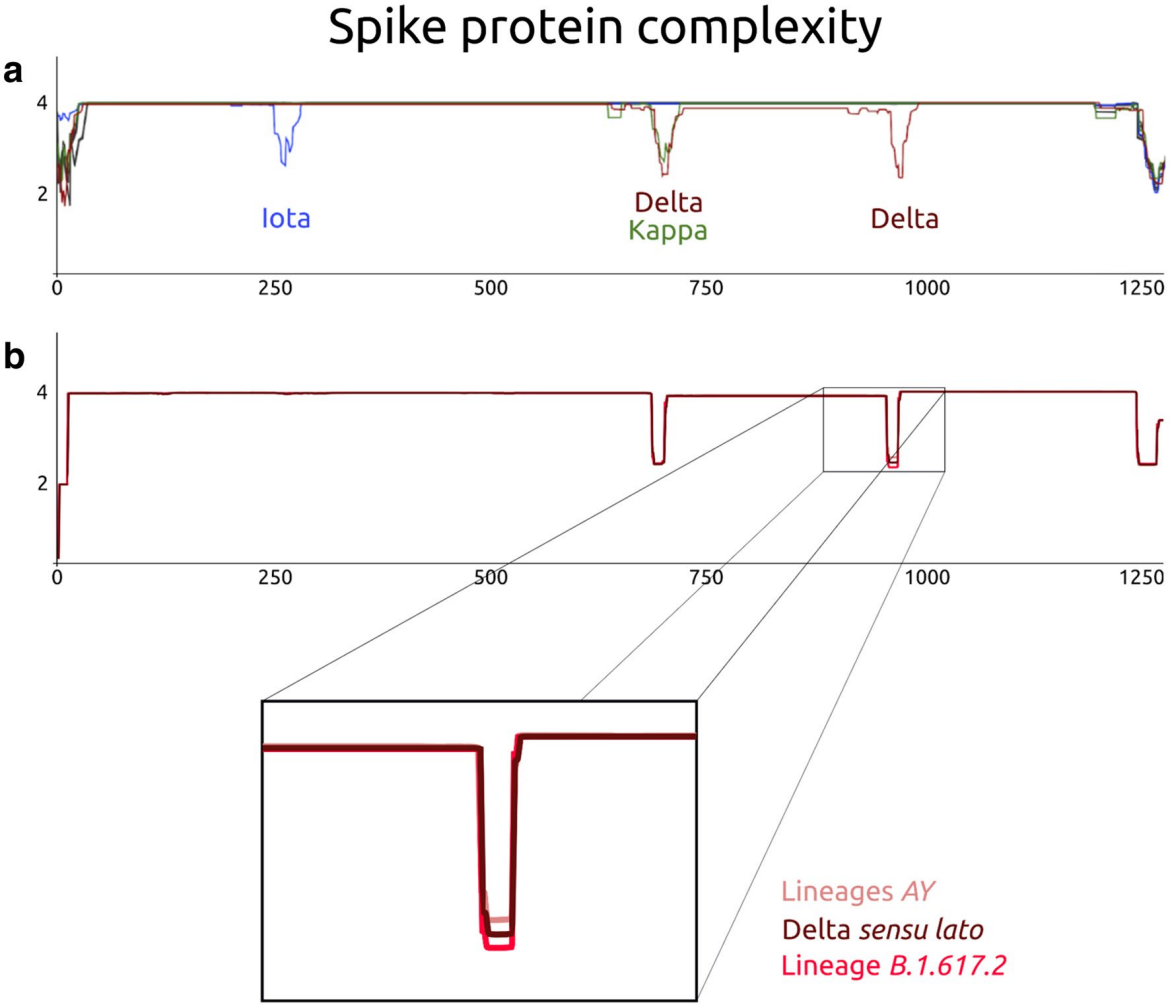
Complexity of the spike proteins of VOIs, VOCs and other SARS-CoV-2. The x axis shows the number of amino acid residues and the y axis shows the complexity level. A) The complexity level of each variant is in a different color: Iota—blue; Delta—dark red; Kappa—green. B) Complexity of the Delta spike proteins. The complexity level of the subsets is in a different color: Delta sensu lato—dark red; Variants AY—salmon pink; lineage B.617.2—bright red.

In this work, we have analyzed a total of 28,231 SARS-CoV-2 whole proteomes (July 17, 2021) and 261,051 spike protein sequences (November 4, 2021) to search for LCRs. As summarized in Figs. 1, 2, and Figure S1, our results indicate that most of the LCRs are present in the viral reference genome and its variants. However, we have detected important differences in the prevalence of these LCRs between the SARS-CoV-2 VOCs and VOIs proteomes.

**Figure 3:**
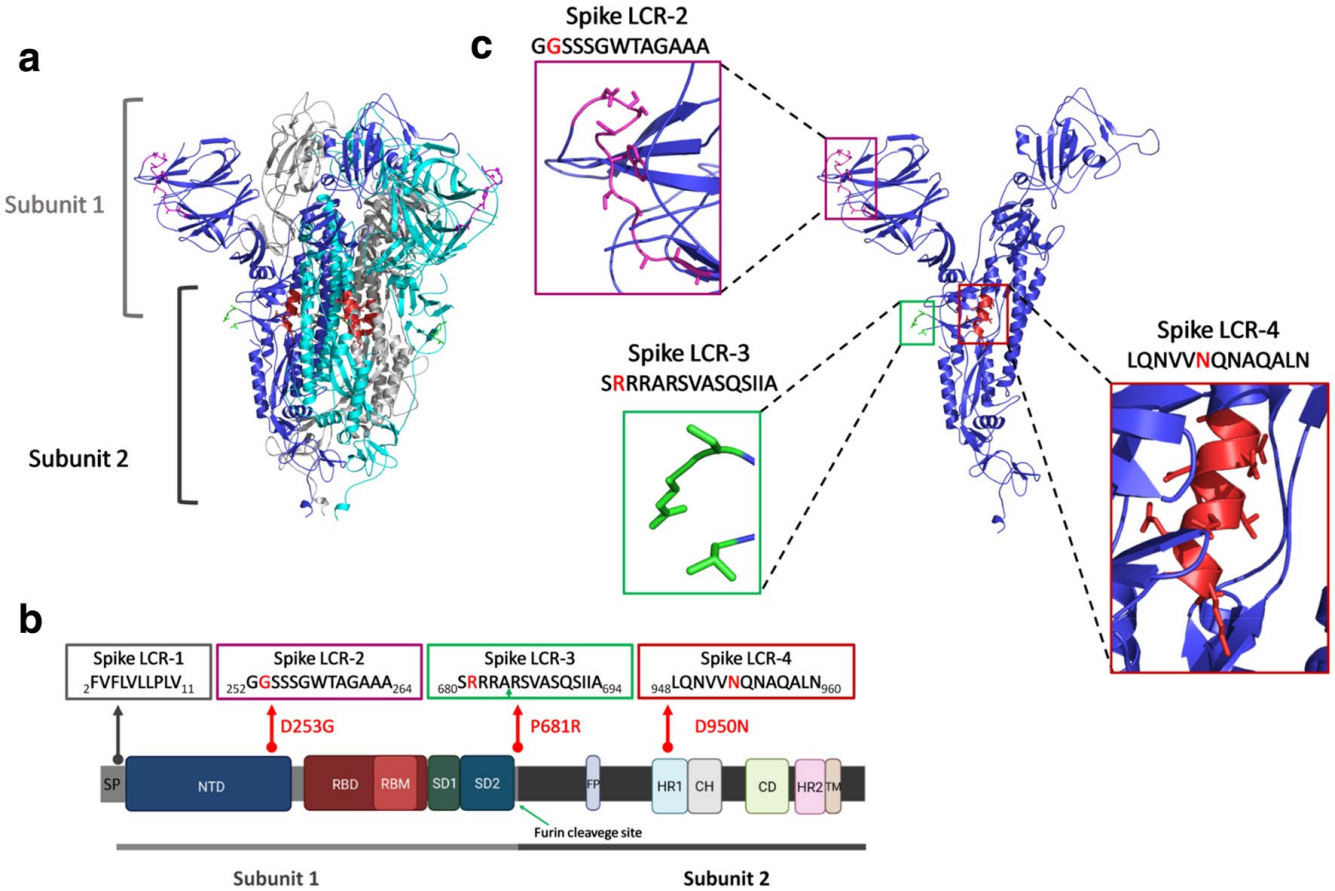
(**a**) The SARS-CoV-2 spike protein three-dimensional structure (by cryo-electron microscopy^24^, PDB code: 7BNM). The structure corresponds to a trimer, where each monomer is represented with a different color. The two subunits that make up each monomer (Subunit 1, also known as Head region, and Subunit 2, or the Stalk region) are indicated. (**b**) Domain organization of the spike protein. The position of each of the LCRs found in this work, together with the mutations present in each variant spike protein are shown. The position of the signal peptide (SP) and Spike LCR-1 are indicated. The green arrow in the Spike LCR-3 box indicates the furin cleavage site. (**c**) Monomer of the spike protein. Close ups of each of the structural regions corresponding to the different LCRs are shown in colored boxes. The sequences of each LCR are represented, with the mutations indicated with a red letter. Protein structures in panels a and c were rendered using PyMOL (The PyMOL Molecular Graphics System, Version 2.0 Schrödinger, LLC.). Panel b was created with BioRender.com. Abbreviations: SP, signal peptide; NTD, N-terminal domain; RBD, receptor-binding domain; RBM, receptor-binding motif; SD1, subdomain 1; SD2, subdomain 2; FP, fusion peptide; HR1, heptad repeat 1; CH, central helix; CD, connector domain; HR2, heptad repeat 2; TM, transmembrane domain.

As shown in Figs. 1 and S1, the Spike LCR-1 formed by the sequence FVFLVLLPLV is present between residues 2 and 11 of all the spike proteins^11^ except for the Iota variant. Here, we report three previously undescribed, highly prevalent, short specific LCRs in the spike proteins of the Delta-, Iota-, and Kappa variants (Spike LCR-2, Spike LCR-3, and Spike LCR-4) (Figs. 1, 2 and Figure S1). In this work we have named each LCR according to the following rules: the first word of the name corresponds to the protein in which the LCR is located, and the number corresponds to its position in each of the SARS-CoV-2 proteins (Table S3). The overall properties of the LCR’s described here, are summarized in Table 1. Figure 3a displays the actual location of these LCRs in a spike protein 3D structure (PDB ID: 7BNM). The LCR which we have named Spike LCR-2 (Fig. 3) is located between the residues 252 and 264 of the N-terminal domain (NTD) of the Iota variant spike protein (Fig. 3c). The sequence of this LCR is GGSSSGWTAGAAA (Fig. 3b and Table 1), and it is present in 79.87% of the Iota variants from the proteomes sample. In contrast, this LCR is absent in the Eta-, and Kappa variants (Fig. S1), and its prevalence in other VOCs, VOIs, and other SARS-CoV-2 samples is below 3% (Fig. S1). Analysis of the spike protein sequences database yielded similar results, indicating that this LCR is present in 99.02% of Iota variants and practically absent in others.

**Table 1:** Spike proteins LCRs reported in this work for the Delta, Iota and Kappa variants.

Name	Spike LCR	~ Start position	~ End position	Variants	Nº of amino acids *	Molecular Weight *	Theoretical pI *	Aliphatic index *	Grand average of hydropathicity *
Spike LCR-1	FVFLVLLPLV	2	11	All (except for Iota)	10	1159.52	5.52	243.00	3.180
Spike LCR-2	GGSSSGWTAGAAA	252	264	Iota	13	1079.09	5.52	30.77	0.123
Spike LCR-3	SRRRARSVASQSIIA	680	694	Delta, Kappa	15	1657.90	12.48	91.33	− 0.407
Spike LCR-4	LQNVVNQNAQALN	948	960	Delta	13	1425.56	5.52	120.00	− 0.377

The position of each LCR in the spike sequence is indicated. The first letter of the name corresponds to the protein in which the LCR is located, and the number corresponds to its position in each of the SARS-CoV-2 proteins. (*) This information was derived from https://www.expasy.org/resources/protparam.

The Spike LCR-3 (Delta-Kappa prevalent) is positioned between residues 680 and 694 in the Delta- and Kappa spike protein variants (Fig. 3b). Its sequence is the polybasic, conserved 15 amino acid segment SRRRARSVASQSIIA (Table 1), that is located precisely in the furin cleavage site in the S1 C-terminus, whose tertiary structure has not been visualized due to its inherent flexibility^24^ (Fig. 3c). In the proteome sample we have analyzed, this LCR is found in 99.19% of the Delta variants (Fig. S1). As shown in Fig. 2, the complexity value of this region in the Delta- and Kappa variants is significantly lower in comparison with the rest of the protein; however, a small number of the Delta sequences (39/4830) do not surpass our cutoff value due to the presence of amino acid substitutions that raise the complexity value of the regions. Analysis of the spike protein sequences database shows that the Spike LCR-3 is present in 99.44% of Delta variants (B.1.617.2) but appears only in 0.52% of other variants.

The other LCR, or Spike LCR-4, has the conserved 13-aa polar-rich sequence LQNVVNQNAQALN, and is located between residues 946 and 958 of the spike protein of the highly transmissible Delta variant. It is found in an alpha-helix rich domain (HR1) (Fig. 3b) that is part of the spike protein S2 stalk region (Fig. 3a). In the proteome dataset, this low complexity region is present in practically every Delta variant; only 1.7% do not surpass the LCR cutoff value defined here (Fig. 1, Fig. S1). In the Beta-, Eta-, and Kappa variants analyzed here, this LCR is completely absent (Fig. S1), whereas in the other SARS-CoV-2 categories, its prevalence is below 2% (Fig. S1). The analysis of the spike protein set shows that the Spike LCR-4 is present in 98.13% of Delta variants (B.1.617.2) and is missing in 99.88% of the other variants in our sample (Fig. 2b and Supplementary Table S1).

In the Alpha variant sequences analyzed here, the NSP3 LCR-3 (Fig. S1) is missing in 98.95% of the proteomes. The Lambda NSP3 LCR-4 (Fig. S1) is absent in 98.84% of the analyzed proteomes.

The available information does not allow any inference on the possible geographical distribution of the different SARS-CoV-2 spike proteins where the LCRs reported here are located (Table S2).

## Discussion and conclusions

LCRs are found in a broad spectrum of proteins and appear to contribute to the antigenic variability in both viral and cellular pathogen populations. Although polymerase slippage events may be involved^25–27^, the mechanisms that produce viral LCRs are poorly understood. The processes that lead to the LCR preservation in highly streamlined genomes, such as those of most RNA viruses, are not well understood, and their tempo and mode of evolution remain open issues. However, the conservation of the two small LCRs (Spike LCR-3, Spike LCR-4) reported here in the rapidly spreading Delta variant suggests that together with mutations found in the nucleocapsid^28^ they may be part of its hallmark traits. Accordingly, a detailed analysis of their frequency and phenotypic significance may contribute to the understanding of the origin of this variant’s increased transmissibility. Dozens of Delta subvariants have been reported throughout the world since the original submission of this paper. All these subvariants have different defining mutations^29^ and their properties are still being investigated. Our analyses of the spike proteins of these variants show that a highly significant percentage are endowed with (Table S1) the same LCRs described in the original SARS-CoV-2 Delta spike protein itself^30,31^.

The Spike LCR-1 (FVFLVLLPLV) is a highly hydrophobic region that consists of helix-forming residues, including phenylalanine, valine and leucine, and it is the major component of the signal peptide (amino acids 1–13) located upstream of the N-terminus domain^32,33^ (Fig. 3). In the lumen of the endoplasmic reticulum this signal peptide plays a key role in guiding the spike protein to its membrane location by cellular signal peptidases^34^.

As noted above, the Kappa/Delta Spike LCR-3 and the Delta Spike LCR-4 regions are located in the spike S1 and S2 subunits, respectively. The mutation P681R detected in the Spike LCR-3 (SRRRARSVASQSIIA) (Fig. 3) at the furin cleavage site increases the polybasic nature of this region, which could augment its affinity with the furin protease^35^. In vitro experiments and SARS-CoV-2 infections in animal models have demonstrated that the P681R mutation enhances both the fusogenicity and pathogenicity of the virus^36^. The phylogenetic relation between the Kappa- and Delta variants, both of which are part of the lineage B.1.617^37,38^, very likely explains the presence of these two mutations in both the Delta- and the Kappa Spike LCR-3 (Figs. 1 and S1).

The ectodomain of the SARS-CoV-2 spike protein is endowed with two heptad repeat motifs (HR1 and HR2) which are involved in cell fusion, which is a key step in viral entry^39,40^. The Spike LCR-4 (LQNVVNQNAQALN) includes charged-neutral, polar (asparagine and glutamine) and hydrophobic amino acids (leucine, valine, and alanine), which are typical of heptad repeat motifs. The interaction of HR1 and HR2 leads to the formation of a six-helical bundle that mediates cell fusion^39^. Accordingly, it is possible that the asparagine (N) of the mutation D950N (Fig. 3) of the Spike LCR-4 may enhance the stabilization of the post-fusion hairpin conformation, since the conservation of the N and Q residues of HR1 is known to play an important role in the arrangement of hydrogen-bonding zippers that force HR2 to adopt its final conformation in SARS-CoV^40^. The structural relevance of this region has been demonstrated by studies with other RNA viruses, in which the use of fusion inhibitors that disrupt HR1-HR2 conformational changes, are known to limit viral entry^41,42^.

Although there may be minuscule variations in the LCRs length and/or amino acid composition, the segments described in this work fall well within the low complexity category and open the possibility that their biased composition may confer adaptive advantages to the Delta variant. For instance, the polybasic Spike LCR-3, which includes several arginines in its N-terminus, is a highly conserved sequence located precisely in the furin cleavage site at spike S1/S2, which is essential for membrane fusion, and plays a key role in viral infection and transmission^42–44^.

The use of the stringent cut-off value used here (W = 12, K1 = 1.9, K2 = 2.1) shows that, except for a limited number of sequences of the Spike LCR-3 and the Spike LCR-4, these two LCRs are extremely prevalent (99.19% and 98.3% of all proteomes, and 99.44% and 98.13 of the subset of spike protein sequences). Although they display the biological traits of typical low complexity regions (Fig. 2), the multiple sequence alignments (Supplementary file 1 and 2) of the sequences that escape our cutoff values show single point mutations within these LCRs. These single-amino acid substitutions increase the complexity of the fragments and prevent their detection by the methodology employed here.

The SARS-CoV-2 Delta variant was detected in the late 2020^37^, and the proteomic traits described here may contribute together with other features to explain in part its rapid worldwide expansion. The role of LCRs in enhancing sequence variability in surface proteins of viral and cellular pathogens has been postulated^5,9,11^. The conservation of the position and the sequence of two LCRs (Spike LCR-3 and Spike LCR-4) in the Delta variant we have described here highlights the importance of LCRs, which might lead to the evolution and development of new functions or the improvement of existing ones.

Simple repeats have been shown to lead to variations in genome size in cellular systems^45^. However, although compositionally biased sequences in SARS-CoV-2 are quite ubiquitous in most of the coronaviral proteins (Fig. 1 and S1), they do not contribute significantly to the increase of its genome size. In contrast, we hypothesize that the high conservation of the two LCRs in the Delta spike protein suggests that, together with the seven mutations present in this variant, they are part of the phenotypic traits associated with its high infectivity. Laboratory studies are required to confirm the possibility that the presence of compositionally biased segments in the Delta variant spike protein may be related to increased transmission, which is part of the defining features of VOCs and VOIs^46–48^.

## Methods

### SARS-CoV-2 proteome sequences

To retrieve a list of proteomes meeting the requirements to be considered as input to the pipeline (https://github.com/abelardoacm/SARS-COV2_LCRs), we downloaded metadata of all the sequences available on the China National Center for Bioinformation web portal (https://ngdc.cncb.ac.cn/news/85) on July 17, 2021. The entries were filtered, keeping only those that corresponded to complete proteomes (Nuc. Completeness = Complete), with high sequence quality (Sequence Quality = High) available in NCBI GenBank (Data Source = GenBank). The proteome sample size per variant was limited to a maximum of 4,000 sequences, a figure comparable to the numbers of the Alpha- and Delta samples analyzed here and included multiple geographical regions (217 locations from 64 countries) that were sampled between January 20, 2020 and July 17, 2021. A subset was made for each variant classified either as a VOC (Alpha n = 3903; Beta n = 384; Gamma n = 4000; and Delta n = 4830) or as a VOI (Eta n = 363; Iota n = 4000; Kappa n = 115; and Lambda n = 259). We have also included proteomes from a random sampling using the R *sample{base}* function, of 10,377 non-VOC/VOI that met the same quality criteria and were classified as "Others SARS-CoV-2" (Others SARS-CoV-2 n = 10,377). Proteomes were downloaded using NCBI batch entrez. Accessions with empty fields in their metadata were discarded, leaving a total of 28,231 proteome files (Supplementary file 3).

### SARS-CoV-2 spike protein database

To broaden our analyses, we also included 261,051 spike protein sequences downloaded from the NCBI Virus database (www.ncbi.nlm.nih.gov/labs/virus/vssi/#/ surface glycoprotein) available up to November 4, 2021. In this database 6,514 sequences correspond to Delta variants (3,269 B.1.617.2 and 3,235 subvariant AY), and 254,537 correspond to other variants (Table S1).

### Detection of low complexity regions (LCRs)

To search for the LCRs in the sample, the SEG^49^ algorithm was used with W = 12, K1 = 1.9, K2 = 2.1 parameters, which are slightly stricter than the default values (W = 12, K1 = 2.2, K2 = 2.5). The pipeline "SARS-COV2_LCRs" was built to couple annotation data from genomic GenBank files with SEG output files and locate and identify LCRs within each genome.

A "genomic features" csv-file containing coordinates for both genes and proteins was prepared, which served as a template to create a proteomic fasta enriched with location information. All the PERL and R scripts we have employed are available at https://github.com/abelardoacm/SARS-COV2_LCRs.git.

Once all LCRs were identified within all proteomes and spike protein sequences in our sample, their frequency was calculated using an R script (Fig. S1). From this analysis, LCRs of interest were selected based on their high prevalence in each variant proteome dataset (Table S1). Subsequently, a LCRs of interest presence matrix was calculated by an R script and used as input to plot the total counts per variant and number of versions per low complexity region (Fig. 2). The amino acid composition of the 4830 Delta spike sequences was analyzed with a multiple sequence alignment built with MUSCLE^50^ v3.8.1551, followed by an amino acid Logo representation (Fig. S2) made with the WebLogo 3 program (http://weblogo.threeplusone.com/create.cgi^51^).

## Supplementary Information

Supplementary Figure S1.

Supplementary Figure S2.

Supplementary Tables.

Supplementary Information 1.

Supplementary Information 2.

Supplementary Information 3.
