# Supplementary figures and images for: Two short low complexity regions (LCRs) are hallmark sequences of the Delta SARS-CoV-2 variant spike protein
Source: Sci Rep. 2022 Jan 18;12:936. doi: 10.1038/s41598-022-04976-8 (PMC8766472; doi:10.1038/s41598-022-04976-8)

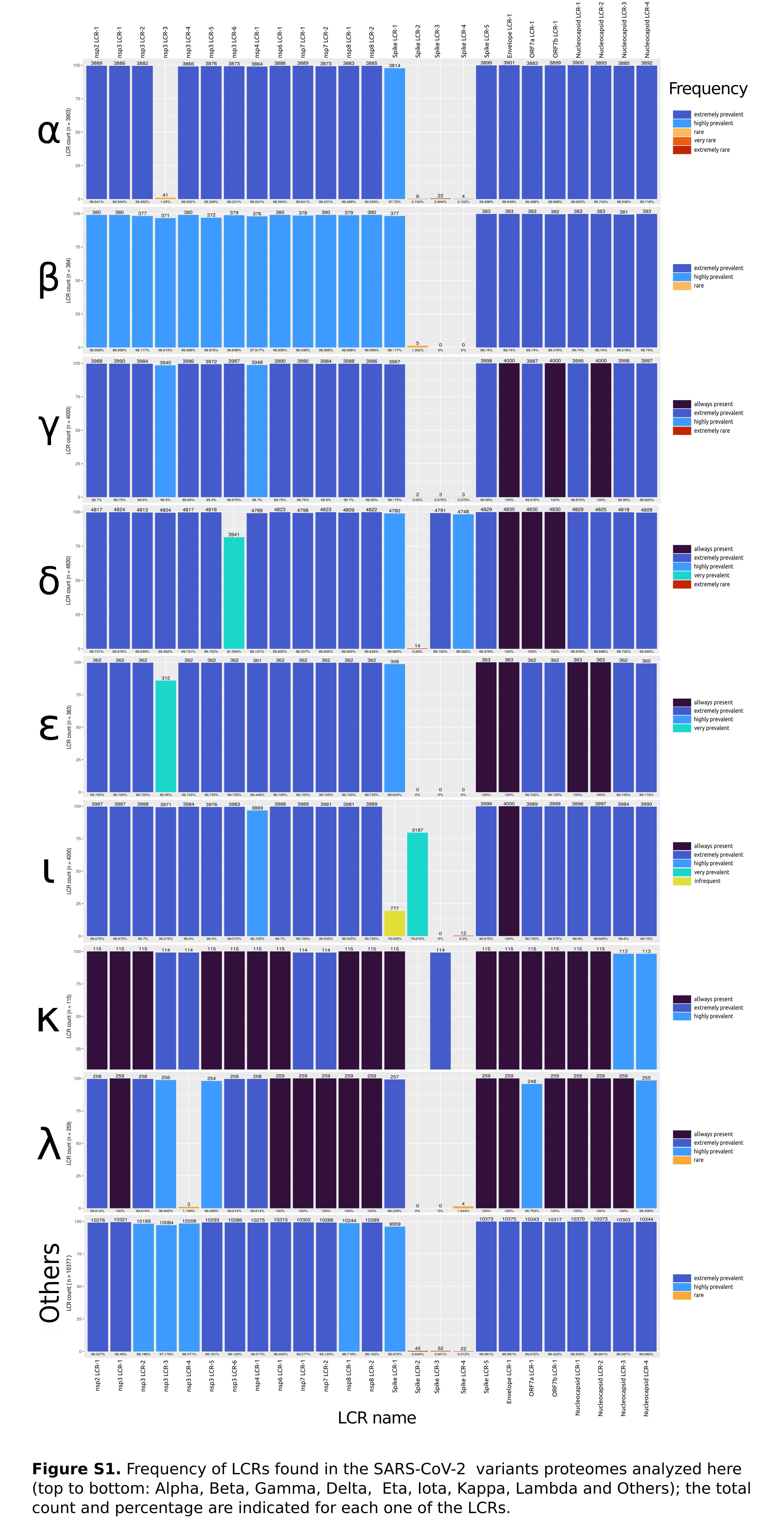

Supplement: Supplementary file 1 — Supplementary Figure S1. [file 41598_2022_4976_MOESM1_ESM.png]

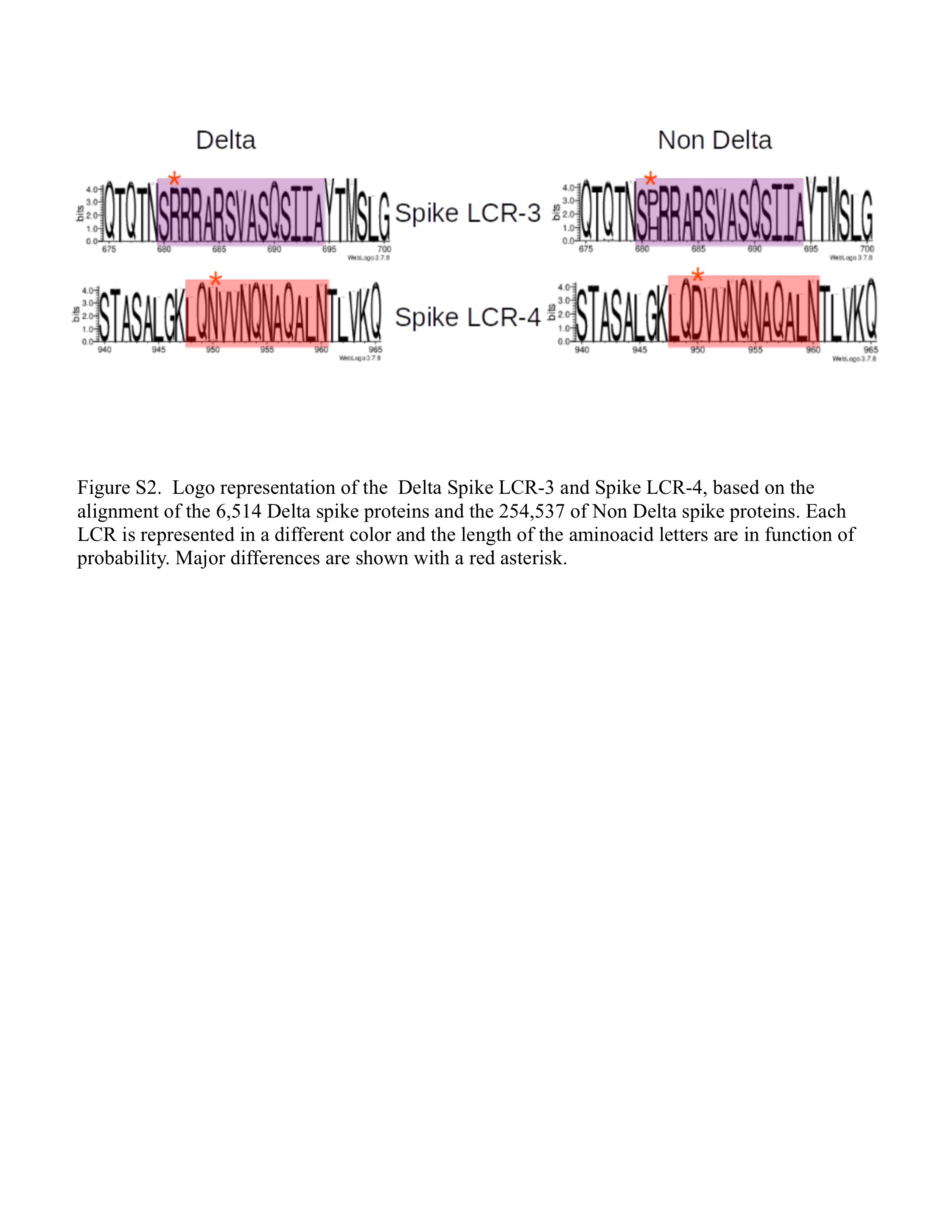

Supplement: Supplementary file 2 — Supplementary Figure S2. [file 41598_2022_4976_MOESM2_ESM.tiff]
