# Supplementary material for: Two short low complexity regions (LCRs) are hallmark sequences of the Delta SARS-CoV-2 variant spike protein
Source: Sci Rep. 2022 Jan 18;12:936. doi: 10.1038/s41598-022-04976-8 (PMC8766472; doi:10.1038/s41598-022-04976-8)
Supplement: Supplementary file 3 — Supplementary Tables. [file 41598_2022_4976_MOESM3_ESM.docx]

**Table S1** Spike LCR-3 and LCR-4 prevalence of Delta and non-Delta variants

| **Lineage** | **Number of spike sequences** | **Spike LCR-3 frequency** | **Spike LCR-3**  **percentage** | **Spike LCR-4**  **frequency** | **Spike LCR-4**  **percentage** |
| --- | --- | --- | --- | --- | --- |
| AY.1 | 4 | 4 | 100 | 4 | 100 |
| AY.2 | 5 | 5 | 100 | 5 | 100 |
| AY.3 | 97 | 97 | 100 | 61 | 62.88659794 |
| AY.3.1 | 70 | 70 | 100 | 45 | 64.28571429 |
| AY.4 | 3 | 3 | 100 | 3 | 100 |
| AY.4.2 | 1 | 0 | 0 | 0 | 0 |
| AY.4.3 | 33 | 33 | 100 | 33 | 100 |
| AY.5 | 4 | 4 | 100 | 3 | 75 |
| AY.10 | 2 | 2 | 100 | 2 | 100 |
| AY.12 | 2 | 2 | 100 | 2 | 100 |
| AY.13 | 21 | 21 | 100 | 17 | 80.95238095 |
| AY.14 | 10 | 10 | 100 | 9 | 90 |
| AY.15 | 1 | 1 | 100 | 1 | 100 |
| AY.16 | 1 | 1 | 100 | 1 | 100 |
| AY.19 | 1 | 1 | 100 | 1 | 100 |
| AY.20 | 14 | 14 | 100 | 10 | 71.42857143 |
| AY.21 | 2 | 2 | 100 | 2 | 100 |
| AY.23 | 5 | 3 | 60 | 3 | 60 |
| AY.24 | 14 | 14 | 100 | 9 | 64.28571429 |
| AY.25 | 379 | 378 | 99.73614776 | 230 | 60.68601583 |
| AY.26 | 73 | 73 | 100 | 42 | 57.53424658 |
| AY.28 | 2 | 2 | 100 | 2 | 100 |
| AY.29 | 18 | 18 | 100 | 18 | 100 |
| AY.29.1 | 1 | 1 | 100 | 1 | 100 |
| AY.32 | 3 | 3 | 100 | 3 | 100 |
| AY.33 | 10 | 10 | 100 | 10 | 100 |
| AY.34 | 4 | 4 | 100 | 4 | 100 |
| AY.35 | 2029 | 2027 | 99.90142928 | 1886 | 92.9521932 |
| AY.36 | 2 | 2 | 100 | 2 | 100 |
| AY.37 | 28 | 28 | 100 | 25 | 89.28571429 |
| AY.39 | 45 | 45 | 100 | 24 | 53.33333333 |
| AY.39.1 | 2 | 2 | 100 | 2 | 100 |
| AY.41 | 1 | 1 | 100 | 1 | 100 |
| AY.42 | 6 | 6 | 100 | 6 | 100 |
| AY.43 | 86 | 84 | 97.6744186 | 32 | 37.20930233 |
| AY.44 | 183 | 181 | 98.90710383 | 154 | 84.15300546 |
| AY.45 | 3 | 3 | 100 | 2 | 66.66666667 |
| AY.46 | 6 | 6 | 100 | 6 | 100 |
| AY.46.1 | 1 | 1 | 100 | 1 | 100 |
| AY.46.4 | 3 | 3 | 100 | 3 | 100 |
| AY.47 | 70 | 70 | 100 | 52 | 74.28571429 |
| Total AY | 3245 | 3235 | 99.69183359 | 2717 | 83.72881356 |
| Total B.1.617.2 | 3269 | 3252 | 99.47996329 | 3208 | 98.13398593 |
| Total Delta sensu lato | 6514 | 6487 | 99.58550814 | 5925 | 90.95793675 |
| Total Non Delta | 254537 | 1367 | 0.5370536 | 301 | 0.118253 |
| Total variants | 261051 |  |  |  |  |

**Table S2** Geo-locations of Delta Spike LCRs.

| **Country** | **Delta**  **frequency** | **Spike LCR-3 frequency** | **Spike LCR-3**  **percentage** | **Spike LCR-4**  **frequency** | **Spike LCR-4**  **percentage** |
| --- | --- | --- | --- | --- | --- |
| Bahrain | 6 | 6 | 100 | 4 | 66.66 |
| Bangladesh | 4 | 4 | 100 | 4 | 100 |
| Egypt | 67 | 67 | 100 | 67 | 100 |
| India | 236 | 234 | 99.15 | 230 | 97.45 |
| Japan | 27 | 27 | 100 | 27 | 100 |
| Myanmar | 2 | 2 | 100 | 2 | 100 |
| USA | 6168 | 6139 | 99.52 | 5587 | 90.58 |
| Uzbekistan | 4 | 4 | 100 | 3 | 75 |

**Table S3**. LCRs present in the 28,231 complete genomes of SARS-CoV-2 analyzed in this work.

| **LCR name** | **Gene** | **Protein** | **Start position (aa)** | **End position (aa)** | **Sequence** |
| --- | --- | --- | --- | --- | --- |
| nsp2 LCR-1 | ORF1ab_polyprotein | nsp2* | 742 | 760 | LEGETLPTEVLTEEVVLKT |
| nsp3 LCR-1 | ORF1ab_polyprotein | nsp3* | 926 | 943 | PPDEDEEEGDCEEEEFEP |
| nsp3 LCR-2 | ORF1ab_polyprotein | nsp3* | 970 | 986 | QPEEEQEEDWLDDDSQQ |
| nsp3 LCR-3 | ORF1ab_polyprotein | nsp3* | 998 | 1005 | QTTTIQTI |
| nsp3 LCR-4 | ORF1ab_polyprotein | nsp3* | 1237 | 1255 | EEVTTTLEETKFLTENLLL |
| nsp3 LCR-5 | ORF1ab_polyprotein | nsp3* | 2172 | 2183 | FFTLLLQLCTFT |
| nsp3 LCR-6 | ORF1ab_polyprotein | nsp3* | 2546 | 2558 | KSKCEESSAKSAS |
| nsp4 LCR-1 | ORF1ab_polyprotein | nsp4* | 3043 | 3057 | ISASIVAGGIVAIVV |
| nsp6 LCR-1 | ORF1ab_polyprotein | nsp6* | 3583 | 3599 | LLLTILTSLLVLVQSTQ |
| nsp7 LCR-1 | ORF1ab_polyprotein | nsp7* | 3869 | 3885 | SVVLLSVLQQLRVESSS |
| nsp7 LCR-2 | ORF1ab_polyprotein | nsp7* | 3912 | 3920 | VSLLSVLLS |
| nsp8 LCR-1 | ORF1ab_polyprotein | nsp8* | 3973 | 3989 | SEVVLKKLKKSLNVAKS |
| nsp8 LCR-2 | ORF1ab_polyprotein | nsp8* | 4040 | 4052 | LDNDALNNIINNA |
| Spike LCR-1 | Surface glycoprotein | Spike | 2 | 11 | FVFLVLLPLV |
| Spike LCR-2 | Surface glycoprotein | Spike | 252 | 264 | GGSSSGWTAGAAA |
| Spike LCR-3 | Surface glycoprotein | Spike | 678 | 692 | SRRRARSVASQSIIA |
| Spike LCR-4 | Surface glycoprotein | Spike | 946 | 958 | LQNVVNQNAQALN |
| Spike LCR-5 | Surface glycoprotein | Spike | 1231 | 1253 | MLCCMTSCCSCLKGCCSCGSCCK |
| Envelope LCR-1 | E | Envelope | 17 | 39 | VLLFLAFVVFLLVTLAILTALRL |
| ORF7a LCR-1 | ORF7a | ORF7a | 3 | 14 | IILFLALITLAT |
| ORF7b LCR-1 | ORF7b | ORF7b | 9 | 30 | FYLCFLAFLLFLVLIMLIIFWF |
| Nucleocapsid LCR-1 | N | Nucleocapsid | 175 | 208 | GSRGGSQASSRSSSRSRNSSRNSTPGSSRGTSPA |
| Nucleocapsid LCR-2 | N | Nucleocapsid | 211 | 230 | AGNGGDAALALLLLDRLNQL |
| Nucleocapsid LCR-3 | N | Nucleocapsid | 236 | 249 | GKGQQQQGQTVTKK |
| Nucleocapsid LCR-4 | N | Nucleocapsid | 361 | 379 | KTFPPTEPKKDKKKKADET |

*The start and end position of these proteins are derived from the polyprotein 1ab, not from the non-structural protein itself.
